# Characterization of an A-Type Muscarinic Acetylcholine Receptor and Its Possible Non-neuronal Role in the Oriental Armyworm, *Mythimna separata* Walker (Lepidoptera: Noctuidae)

**DOI:** 10.3389/fphys.2020.00400

**Published:** 2020-04-30

**Authors:** Shumin Lü, Ming Jiang, Xing Tian, Shanwang Hong, Junwei Zhang, Yalin Zhang

**Affiliations:** ^1^Key Laboratory of Plant Protection Resources and Pest Management, National Ministry of Education, College of Plant Protection, Northwest A&F University, Xianyang, China; ^2^College of Life Sciences, Yan’an University, Yan’an, China

**Keywords:** *Mythimna separata*, A-type mAChR, G protein-coupled receptor, pharmacology, Ca^2+^

## Abstract

Muscarinic acetylcholine receptor (mAChR) regulates many neurophysiological functions in insects. In this report, a full-length cDNA encoding an A-type mAChR was cloned from the oriental armyworm, *Mythimna separata*. Pharmacological properties studies revealed that nanomolar to micromolar concentrations of carbachol or muscarine induced an increase of intracellular Ca^2+^ concentration ([Ca^2+^]_*i*_), with the EC_50_ values of 124.6 and 388.1 nM, respectively. The increases of [Ca^2+^]_*i*_ can be greatly blocked by the antagonist atropine, with an IC_50_ value of 0.09 nM. The receptor mRNA is expressed in all developmental stages, with great differential expression between male and female adults. The tissue expression analysis identified novel target tissues for this receptor, including ovaries and Malpighian tubules. The distribution of Ms A-type mAChR protein in the male brain may suggest the neurophysiological roles that are mediated by this receptor. However, the receptor protein was found to be distributed on the membranes of oocytes that are not innervated by neurons at all. These results indicate that Ms A-type mAChR selectively mediates intracellular Ca^2+^ mobilization. And the high level of receptor protein in the membrane of oocytes may indicate a possible non-neuronal role of A-type mAChR in the reproductive system of *M. separata*.

## Introduction

Acetylcholine (ACh) is the predominant excitatory neurotransmitter of the sensory neurons and interneurons within the central nervous system (CNS) of insects (Martin and Krantz, 2014). Mutations in both choline acetyltransferase and acetylcholinesterase have behavioral and physiological effects and can cause lethality (Greenspan, 1980; Greenspan et al., 1980). ACh has been known to exert its physiological function through the ionotropic nicotinic ACh receptors (nAChRs), or the metabotropic muscarinic ACh receptors (mAChRs). These roles, subtypes, and molecular mechanism of nAChRs in insects have been widely documented but, in contrast, the characterization and identification of mAChRs in invertebrates have proceeded slowly (Blake et al., 1993). The insect mAChRs have been less well studied (Honda et al., 2007; Collin et al., 2013). mAChR is a member of the G protein-linked receptor superfamily which contains seven putative transmembrane domains, and can activate several distinct signal transduction pathways by interacting with guanine nucleotide binding proteins (G proteins) (Caulfield, 1993). In mammals, five mAChR subtypes (m1–m5) have been identified pharmacologically, the m1, m3, and m5 coupled to members of the G_*q/*__11_ family, activating phospholipase C and increasing the intracellular calcium level, and the m2 and m4 receptors preferentially coupled to members of the G_*i/*__0_ family, leading to a decrease of intracellular cAMP (Eglen, 2005; Giglio and Tobin, 2009; Bubser et al., 2012). Insect mAChRs do not entirely fit the vertebrate mAChR classification scheme. They are divided into three subfamilies (A-, B-, and C-type) (Collin et al., 2013; Ren et al., 2015; Xia et al., 2016). These results verify the previous research that three candidate mAChRs were found in *Drosophila* by informatics analysis from the complete gene set (Brody and Cravchik, 2000; Yoshihara et al., 2001). The first insect mAChR (CG4356, later identified as an A-type mAChR) has been identified from the fruit fly *Drosophila melanogaster*; activation of this *Drosophila* receptor causes the accumulation of inositol phosphates (Onai et al., 1989; Shapiro et al., 1989; Blake et al., 1993). And the ant *Polyrhachis vicina* mAChR was identified and proposed to be involved in obtaining and integrating the visual and olfaction information in the nervous system (Lü et al., 2011). Then, two types of mAChRs (A-type and B-type) have been cloned from *D. melanogaster* and *Tribolium castaneum*, and the respective A- and B-type orthologs have been identified using bioinformatics from the insects with a sequenced genome. A-type mAChR could be activated by agonists ACh and muscarine, and inhibited by the antagonist atropine and 3-quinuclidinyl-benzilate (QNB); it is coupled to G_*q/*__11_, and is pharmacologically very similar to the mammalian m1–m5 mAChRs. The B-type is also activated by ACh, but has a 1000-fold lower sensitivity to muscarine, and was insensitive for atropine and other classical mAChR antagonists, it is coupled to G_*i/*__0_ resulting in a decrease of the intracellular cAMP concentration (Collin et al., 2013; Ren et al., 2015). A recent study (Xia et al., 2016) extracted a novel mAChR member from *D. melanogaster*, the C-type mAChR, which could be activated by ACh and the mAChR agonist oxotremorine M to increase intracellular Ca^2+^ levels.

The physiological roles of mAChRs in the insect CNS were studied mainly by pharmacological assays. Stimulation of mAChRs by a muscarinic agonist would result in experience-dependent structural brain plasticity (Ismail et al., 2006). The competitive muscarinic antagonists, scopolamine, and atropine would impair olfactory memory retrieval in honey bee (Gauthier et al., 1994; Lozano and Gauthier, 1998; Lozano et al., 2001). And interference expression of mAChR mRNA in MB would fully inhibit the formation of new aversive olfactory memory in larvae (Silva et al., 2015). These results demonstrate that mAChRs are important contributors in the generation of memories, particularly olfactory memory in insects. Moreover, the application of muscarinic agonists activates central pattern generating circuits for various insect behaviors such as singing behavior in grasshopper (Hoffmann et al., 2007; Heck et al., 2009), rhythmic locomotion in locust (Ryckebusch and Laurent, 1993), as well as chewing and “crawling” movements in *Manduca* larvae (Gorczyca et al., 1991; Trimmer, 1995). Localization of mAChR protein in insect nervous system has been studied in *Drosophila* (Blake et al., 1993; Harrison et al., 1995), *Bombyx mori* (Aizono et al., 1997), and *Manduca sexta* (Clark et al., 2005). These results revealed that mAChRs may play multiple roles in insect nervous system. The presynaptic mAChR mediates inhibition of ACh release coupled to reduction of cAMP levels (mammalian M2-like) (Hue et al., 1989), while the postsynaptic receptors similar to the vertebrate m1or m3 subtypes regulate the spike threshold and excitability of motoneurons and interneurons (Trimmer, 1995). Then the mRNA expression of mAChRs was analyzed by dissecting the head, thorax, and abdomen of *D. melanogaster* and *P. vicina* (Lü et al., 2011). But there was no information about mAChRs expression in the concrete tissues of insects. Previous studies about biological roles of ACh were predominantly focused on its action as a neurotransmitter operating within the nervous system, while little is known about its physiological role in other systems, especially the non-neuronal systems. It is important to determine the tissue expression patterns and the localization of the receptors *in vivo*, thus improving our understanding of physiological function of this receptor.

Components of the ACh signaling cascade, such as cholinesterase and the nAChRs, have been extensively studied as targets for insecticides (Jones and Sattelle, 2010; Dupuis et al., 2012). The major family of insecticides targeting nAChRs are the neonicotinoids which have been deployed for 25 years and are still among the most important insecticides worldwide. However, there are concerns regarding the enhanced resistance to neonicotinoids in pests and possible threats to insect pollinators (notably on bees) as well as to the health of the soil (Ihara et al., 2017; Casida, 2018; Matsuda et al., 2020). Because of the adverse ecological effects, the European Union (EU) and along with some non-EU countries restricted the use of certain neonicotinoid insecticides. Casida (2018) pointed out that the neonicotinoids have had a major impact on pest insect control, but they are increasingly being replaced by newer compounds acting at other biochemical targets. The insect mAChR is evaluated as a potential target for insecticide action, which provides a continuing fascination and challenge to discover new chemicals with no target site cross-resistance against known insecticides (Dick et al., 1997; Honda et al., 2007). Additionally, pharmacological difference between mammalian and insect mAChR could be pivotal in the identification of novel chemistry acting specifically at insect mAChRs (Gross and Bloomquist, 2018). However, no insecticide classes are currently available that target mAChRs. The armyworm *Mythimna separata* is a polyphagous pest of nearly 100 families of more than 300 kinds of food and industrial crops. It is becoming a devastating threat for the production of corn especially in northern China (Feng et al., 2008). Better functional understanding of the mAChR may help us for practical applications in the development of environmentally sustainable pesticides for this pest. However, little information is available as to the expression and pharmacological profiles of mAChR in *M. separata*.

The identification of potential mAChR is decisive for an understanding of the cellular pathways or physiological functions involved in mediating the effects of ACh. The aim of this study is to clone and pharmacological characterize the A-type mAChR from *M. separata* and to investigate its tissue expression by quantitative RT-PCR (qRT-PCR) and immunohistochemistry methods. The findings of this study provide further insight into the role and significance of A-type mAChR in this insect, and strongly suggested the non-neuronal function of A-type mAChR in the female reproductive system of insects.

## Materials and Methods

### Insect and Reagent

*Mythimna separata* larvae were initially obtained from the Biorational Pesticides Research and Development Center, Northwest A&F University, Shaanxi, China, and reared on corn leaves at 25 ± 2°C, 50 ± 5% relative humidity, and a photoperiod of 16 h light: 8 h darkness. The moths were supplied with a 5% honey solution as nutrient. Different developmental stages of *M. separata* were selected and immediately placed into RNAlater (Ambion, Austin, TX, United States) and stored at −70°C until use. For tissue collection, male and female adults at 3 days after eclosion were selected and chilled at 4°C for 30 min to sedate the moths. Then the moths were dissected in the 1 × phosphate buffered saline (PBS), the head, midgut, fat body, Malpighian tubules, ovary, and testis were harvested by micro scalpel and tweezers. Tissues were immersed immediately into RNAlater and stored at −70°C until used for RNA extraction. Carbachol, muscarine, and atropine were purchased from Sigma (St. Louis, MO, United States). All other chemicals of research grade were obtained from commercial sources.

### RNA Isolation and cDNA Synthesis

Total RNA was extracted from different developmental stages and various tissues by using Trizol reagent (Takara, Tokyo, Japan), according to the manufacturer’s directions. Contaminating genomic DNA was removed from the purified RNA by treatment with RQ1 RNase-free DNase1 (Promega, Madison, WI, United States). RNA quantity and purity were assessed by using a NanoDrop spectrophotometer (Thermo Scientific, NanoDrop Products, Wilmington, DE, United States). Single-stranded cDNAs were synthesized from 1 μg of total RNA using a First-Strand cDNA Synthesis Kit with oligo (dT) primer (Takara, Tokyo, Japan).

### Cloning of *M. separata* A-Type mAChR

A pair of degenerate primers F1 and R1 (Table 1) were designed from conserved regions of known A-type mAChRs to amplify a fragment of 1566 bp cDNA encoding *M. separata* A-type mAChR from the head cDNA. PCR amplification was performed in 50 μl reaction volume with the following protocol: 94°C for 3 min, followed by 35 cycles of 94°C for 45 s, 53°C for 45 s, 72°C for 75 s, and a final extension at 72°C for 10 min. The amplified fragment of expected size was purified from agarose gel using the Gel Extraction Kit (Biospin, China), and then subcloned into pGEM-T easy vector (Promega, Madison, WI, United States) for sequencing.

**TABLE 1:** Oligonucleotide primers used for Ms A-type mAChR.

Name	Primer sequence 5′-3′	Expected size	Tm
F1	GAT(A/C/T)TC(C/G)TT(C/T)AAGATCGACAA	1566 bp	53°
R1	GC(A/G)TTGCACA(A/G)GGCGTA(A/G)CA		
3′GSP1	GGGATTGACGAAACAGCCGA	532 bp	56°
3′GSP2	CGTTGTCGGCCATCCTTCTA		
5′GSP1	CTTCTGCCGTTTCTTAGTTTCC	715 bp	60°
5′GSP2	CTATCATCATGGTCGCTCTTCG		
F2	ATGCTCATCGCGCTCAATGA	1806 bp	53°
R2	TCAGTTGTATACTCCTCTGG		
QS1	GCATCCCTGACGAACTGT	154 bp	60°
QA1	ATCGCTTCCCTATTCCTAT		
QS2	CCTGCGTCTGGACTTGGC	107 bp	60°
QA2	CGCGCACGATCTCACGCT		
*Nhe*I-F	CTAGCTAGCATGCTCATCGCGCTCAATGA	1824 bp	55°
*Hin*dIII-R	GGCAAGCTTTCAGTTGTATACTCCTCTGG		

*Oligonucleotides underlined mean the restriction sites for *Nhe*I and *Hin*dIII.*

The remaining 5′ and 3′ cDNA sequences were obtained by nested rapid amplification of cDNA ends (RACE) method by using 5′-Full RACE Kit (Code No. D315, Takara) and 3′-Full RACE Core Set (Code No. D823A, TaKaRa), respectively. For 5′ end of A-type mAChR, the first round of PCR was performed using the gene-specific primer 5′ GSP1 (Table 1) and the universal adaptor primer 5′ outer, the second round was performed using the gene-specific primer 5′ GSP2 (Table 1) and the adaptor primer 5′ inner. PCR amplification was performed in 50 μl reaction volume using Tks Gflex DNA Polymerase (Code No. R060A, Takara), with the following protocol: 94°C for 1 min, followed by 30 cycles of 98°C for 10 s, 60°C for 15 s, 68°C for 1 min. Similarly, for the remaining 3′ sequence, two pairs of primers (3′ GSP1 and 3′ outer, 3′ GSP2 and 3′ inner; Table 1) were used for the first and second round of 3′ RACE PCR, respectively. PCR amplification was performed in 50 μl reaction volume with the following protocol: 94°C for 3 min, followed by 35 cycles of 94°C for 45 s, 56°C for 45 s, 72°C for 1 min, and a final extension at 72°C for 10 min. Gene specific primers (S2, A2; Table 1) were designed to synthesize a cDNA containing the complete ORF. PCR amplification was performed in 50 μl reaction volume with the following protocol: 94°C for 3 min, followed by 35 cycles of 94°C for 45 s, 53°C for 45 s, 72°C for 2 min, and a final extension at 72°C for 10 min. The PCR products were purified and sequenced as described above.

### Sequence and Bioinformatic Analysis

The sequenced fragments were assembled using the Lasergene Sequence Analysis software v. 7.0 (DNASTAR Inc., Madison, WI, United States), then blasted against the non-redundant GenBank nucleotide database using BLASTN^1^ searches to determine gene identity. Open reading frame (ORF) was predicted using the NCBI ORF finder, then the putative protein sequence compared against the non-redundant GenBank protein database using Blastp searches (Altschul et al., 1997) to confirm putative protein identity. The NCBI Conserved Domain Database (CDD) (Marchlerbauer et al., 2011) was used to identify conserved domains. Tools available at the ExPASy^2^ were used to determine putative molecular weights and isoelectric points, the structure and functional motifs of the encoded protein, and the transmembrane segments were predicted with TMHMM servers^3^. The PredictProtein server^4^ was also used to predict the structure and functional motifs of the encoded protein. Sequence alignments were performed by ClustalW^5^ based on the amino acid sequences of the selected mAChRs, and the phylogenetic tree was compiled using MEGA (6.0) software^6^ using the neighbor-joining method. Bootstrap confidence values were calculated with 1000 replicates.

### Construction of Expression Vector

The fragment containing ORF was amplified from the cDNA template using the primers with the restriction site (Table 1). The PCR product was subcloned to pMD-19T simple vector (Takara). A-type mAChR in pMD-19T simple vector and the mammalian expression vector pcDNA3.1 (Invitrogen, San Diego, CA, United States) were digested with *Nhe*I and *Hin*dIII. Restriction fragments were gel purified and ligated into *Nhe*I and *Hin*dIII cut pcDNA3.1. The resultant pcDNA3.1-Ms A-type mAChR Plasmid DNA was prepared with Qiagen Plasmid Maxi Kit (Qiagen) and verified by sequencing.

### Cell Culture and Transfection

HEK 293 cells were grown at 5% CO_2_ in Dulbecco’s modified eagle’s media (D-MEM) (Gibco-Invitrogen, Carlsbad, CA, United States) supplemented with 10% newborn calf serum (Gibco-Invitrogen) at 37°C. For transient expression, cells were plated on six-well plates with a density of 10^5^ cells per well. After being cultured for 18 h, cells were transfected with pcDNA3.1-mAChR using Lipofectamine 2000 reagent (Invitrogen) by a modified calcium phosphate method (Chen and Okayama, 1987). 24 h after transient transfection, the cells were collected and seeded to the 384-well plate with 2 × 10^4^ cells/well density. Functional coupling of expressed receptors to intracellular signaling pathways was then tested 24 h later.

### Calcium Mobilization Assay

[Ca^2+^]_*i*_ was monitored with FLIPR^®^ Calcium 4 Assay Kit (Molecular Devices Corp., Sunnyvale, CA, United States) according to the manufacturer’s instructions. After the cells were transient transfected for 48 h, we added 25 μl loading buffer (prepared according to the manufacturer’s direction) per well for the 384-well plate, and incubated at 37°C with 5% CO_2_ for 1 h and then kept the plates at room temperature for at least 15 min in the dark. After incubation, we transferred the plates directly to FLIPR and begin the calcium assay according to the FLIPR system manual. Excitation wavelength was 485 nm, emission wavelength was 525 nm and measured for 120 s. For agonist analysis, a dilution series of agonist (muscarine and carbachol) dissolved in the HBSS buffer (20 mM HEPES) were added automatically to the cells after detection for 20 s. For antagonist analysis, a dilution series of antagonist were added to the cells and incubated at 37°C with 5% CO_2_ for 15 min, and then kept the plates at room temperature for at least 15 min. 10 μM agonist carbachol was added automatically to the cells. Each experiment was repeated three times. 0.1% DMSO buffer was added to determine non-specific binding. pcDNA3.1-GFP transfected and untransfected HEK 293 cells were used as negative controls.

### Cyclic AMP Determination

The cAMP concentration was determined using the cAMP assay kit (Cisbio Bioassays, Codolet, France) according to the manual. After being transient transfected for 48 h, cells were suspended in 10% FBS/D-MEM and plated on the 384-well plate with a density of 2500 cells/well. Then they were pre-incubated in 1 × Dulbecco’s PBS (D-PBS, Gibco BRL) containing 100 μmol/l of the phosphodiesterase inhibitor isobutylmethylxanthine (IBMX) for 20 min at 37°C. After the pre-incubation, various concentrations of agonists were added and incubated at room temperature for 30 min. For antagonist studies, the stimulations were carried out as above except that the dilution series of antagonists were mixed with 10 μM agonist carbachol. We then added 5 μl cAMP-d2 and 5 μl anti cAMP-Cryptate and incubated it at room temperature for 1 h in the dark. 0.1% DMSO buffer was added to determine non-specific binding. pcDNA3.1-GFP transfected and untransfected HEK 293 cells were used as negative controls. Results are calculated from the 665/620 nm ratio by PHERAstar PLUS (BMG Labtech, Offenburg, Germany) and expressed in Delta F. Each experiment was performed in duplicate and repeated three times.

### Gene Expression Profile Analysis

**FIGURE 1:**
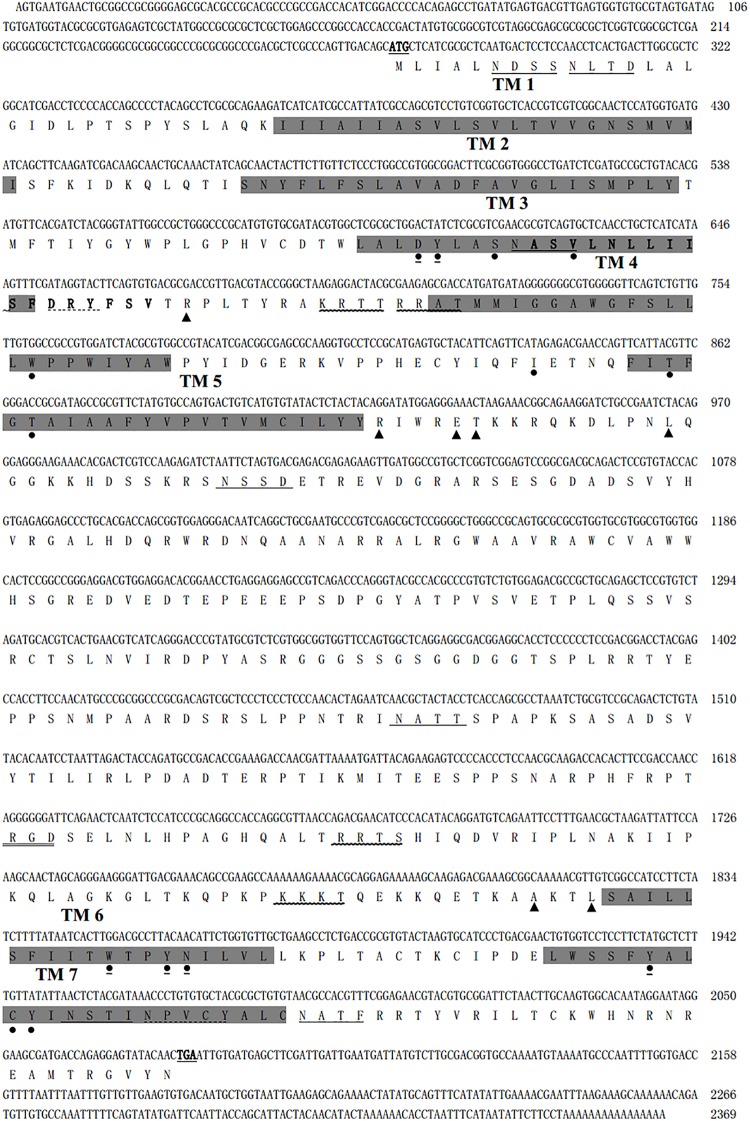
The nucleotide and deduced amino acid sequences of *Mythimna separata* A-type mAChR. The initiating codon ATG and the stop codon TAA are underlined and boldfaced. Seven N-glycosylation consensus sequences were underlined. Seven transmembrane-spanning domains are marked in gray. One cell attachment sequence RGD is double lined. Four cAMP- and cGMP-dependent protein kinase phosphorylation sites were marked with a wavy line. One G-protein coupled receptors signature is boldfaced. A DRY motif and the NPXXY motif are marked with a dotted line. The conserved amino acid residues that are responsible for coupling the mAChRs to G_*q/*__11_ are marked with a triangle. 14 amino acid residues involved in the antagonist QNB binding are marked with a circle. The proposed (“modeled”) binding residues for acetylcholine are marked with a circle and underlined.

Expression profiles of A-type mAChR mRNA for different developmental stages of *M. separata* and in various tissues of adults were analyzed by relative quantification employing real-time RT-PCR. For each experimental series, 10 individuals of developmental stages and tissues from 15 adults of *M. separata* were pooled, and we performed three repeats. The β-actin gene was used as a “housekeeping gene” (Li et al., 2010) to normalize the A-type mAChR transcript levels. Gene specific primers for A-type mAChR (QS1, QA1; Table 1 and Figure 1) and β-actin (QS2, QA2; Table 1) yielded PCR products of 154 and 107 bp, respectively. PCR amplification and fluorescence detection were performed using a Cycler iQ Real-Time PCR detection system (Bio-Rad iQ^TM^5, Hercules, CA, United States) with a SYBR Premix Ex Taq Kit (TaKaRa). Amplification reactions were carried out in a 96-well plate with 25 μl reaction volume, which consisted of 1 μl of cDNA templates, 12.5 μl 2 × SYBRGreen master mix, 1 μl of each specific primer (10 μM), and 9.5 μl double-distilled H_2_O. The following protocol was applied: 95°C for 1 min, 40 cycles of 95°C for 10 s, and 60°C for 30 s, followed by a dissociation curve program from 60 to 95°C with a heating rate of 0.5°C and a continuous fluorescence acquisition. Gel and melting curve analysis were carried out to ensure that there was a single product of the correct size. Runs without a DNA sample (ddH_2_O substitute) were used as the negative control and the reaction was run in triplicate. PCR efficiencies of these two genes were calculated from a standard curve made from a dilution series of a mix of the cDNAs from all samples, and were similar and near to 100%. Relative expression levels were determined using the formula 2^–Δ^ ^Δ^ ^*Ct*^ (Livak and Schmittgen, 2001).

### Generation of Ms A-mAChR Antibody

The protein sequence was antigenic analyzed by the OptimumAntigen^TM^ design tool^7^. We selected the antigenic peptide “CKWHNRNREAMTRGV” located at the C terminal of the predicted Ms A-mAChR protein sequence (585–599). It was also specific in A-mAChRs when compared with B-and C-type. The peptide was run blastp in a protein database^8^ to determine that other proteins (not A-mAChRs) did not contain a similar peptide sequence. The peptide was synthesized as an antigen in the production of affinity-purified New Zealand rabbit polyclonal antibodies. The carrier KLH was conjugated to the beginning of the sequence by a cysteine. Peptide synthesis, conjugation to KLH, antibody production, and affinity purification were performed by GenScript (Piscataway, NJ, United States).

### Immunohistochemistry

The ovaries and heads were dissected from adults at 3 days after eclosion, and immediately fixed in freshly prepared 4% paraformaldehyde in 0.1 M PBS at room temperature for 6 h and then stored in 0.1 M PBS containing 0.2% Triton X-100 overnight; 8 μm cryosections were mounted onto polylysine-coated slides (Boster Company, Wuhan, China) and air dried for 3 h, followed by re-hydration in ddH_2_O for 5 min. Slides were then put in citrate buffer solution for antigen retrieval by high-temperature heat method (98°C for 30 min). SP staining method (Boster Company, Wuhan, China) was performed according to the manufacturer’s directions. Sections were incubated with the anti-Ms A-mAChR antibody (GenScript, Piscataway, NJ, United States), at a concentration of 3 μg/ml in PBS overnight in a humidified chamber at 4°C. After washing three times with 0.1 M PBS, 5 min each time, sections were incubated in the secondary antibody goat anti-rabbit IgG conjugated with biotin (Boster Company, Wuhan, China) at 37°*C* 30 min. Sections were washed in PBS five times, 5 min each time, then stained with diaminobenzidine (DAB) detection kit (Boster Company, Wuhan, China). Substitution of the pre-immune serum for the anti-MsA-mAChR antibody was conducted as the control. Sections were photographed with a LEICA QwinV3 image analysis system (LEICA, Germany).

## Results

### Cloning and Characterization of A-Type mAChR in *M. separata*

**FIGURE 2:**
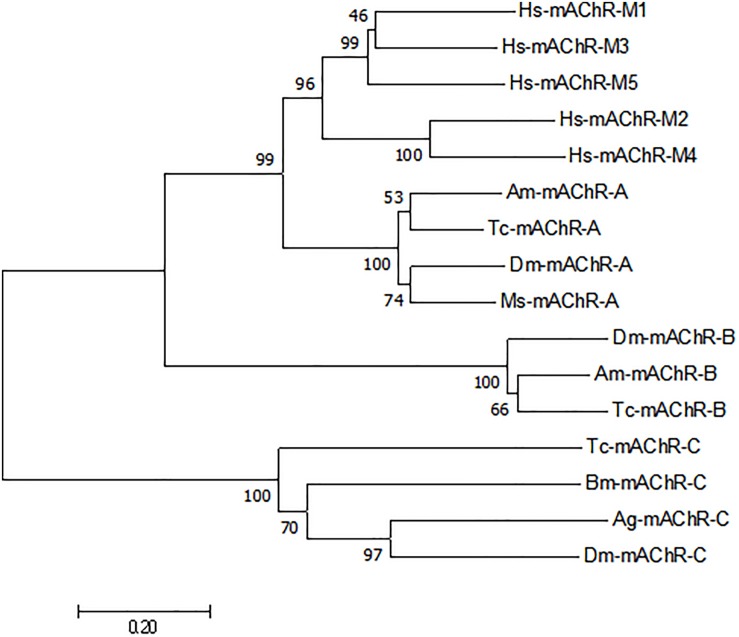
Phylogenetic tree constructed on the basis of overall protein sequences of mAChRs from insects and human m1–m5 mAChRs (released in GenBank). Numbers at branch nodes are percentages of bootstrap confidence values. The phylogenetic tree was constructed by MEGA 7.0, using the neighbor-joining method with bootstrap test with 1000 replicates and a Poisson correction model. *Mythimna separata*, Ms: Ms-mAChR-A KY296116; *Apis mellifera*, Am:Am-mAChR-A XP_395760.4, Am-mAChR-B XP_006558421.1; *Bombyx mori*, Bm: Bm-mAChR-C XP_004924179.1; *Tribolium castaneum*, Tc: Tc-mAChR-C EFA01319.1, Tc-mAChR-A AFJ23966.1, Tc-mAChR-B AFJ23968.1; *Anopheles gambiae*, Ag: Ag-mAChR-C XP_310742.3; *Drosophila melanogaster*, Dm: Dm-mAChR-A AFJ23965.1, Dm-mAChR-B AGE13748.1, Dm-mAChR-C AAF46208.1; *Homo sapiens*, Hs:Hs-mAChR-M1 NP_000729.2, Hs-mAChR-M2 NP_001006633.1, Hs-mAChR-M3 NP_000731.1, Hs-mAChR-M4 NP_000732.2, Hs-mAChR-M5 NP_001307846.1.

A 1566-bp cDNA fragment was obtained by homologous cloning based on the conserved sequences of mAChRs from other insects. The NCBI Blastn running result showed that it was homologous to insect mAChRs. Then a 532-bp fragment of 3′ RACE and a 715-bp fragment of 5′ RACE were obtained from the nested PCR based on the known sequence. The three overlapping fragments were assembled and achieved a 2369 bp full-length cDNA for *M. separata* mAChR (GenBank accession no. KY296116). It contains an ORF of 1806 bp, with a 5′-untranslated region (UTR) of 274 bp and 3′-UTR of 289 bp (Figure 1). Gene specific primers (S2, A2) across ORF were used to confirm the sequence obtained. Sequenced results reveal that it entirely overlapped with the assembled mAChR cDNA. The ORF encodes a protein of 602 amino acid residues with a predicted molecular mass of 67.4 kDa and an isoelectric point of 9.42. Comparison of this protein sequence in GenBank employing Blastp revealed a high degree of similarity (>65%) with *D. melanogaster* and *T. castaneum* A-type mAChR. We designated it as Ms A-mAChR. Transmembrane-spanning domains prediction analysis revealed the presence of seven hydrophobic domains as membrane-spanning segments (TM1–TM7, Figure 1). Seven *N*-glycosylation consensus sequence NX[ST]X: 6–9 NDSS, 10–13 NLTD, 115–118 NASV, 244–247 NSSD, 397–400 NATT, 560–563 NSTI, 572–575 NATF, four cAMP- and cGMP-dependent protein kinase phosphorylation sites [RK]X[ST]: 141–144 KRTT, 145–148 RRAT, 466–469 RRTS, 499–502 KKKT, and one cell attachment sequence 449–451 RGD were found in the protein sequence (Figure 1). One G-protein coupled receptor signature (position 116-132) was found in TM3 and IL2 (Figure 1); 14 amino acid residues forming a binding pocket for the antagonist QNB (Haga et al., 2011; Collin et al., 2013) are distributed in the TM3–TM7 transmembrane helices of the receptor (marked with a circle in Figure 1). The proposed (“modeled”) 6 binding amino acid residues for ACh (Haga et al., 2011) are also found in this receptor (Figure 1). The conserved DRY (Asp 127-Arg 128-Tyr 129) residues at the IL2 and a NPXXY motif in TM 7, both of which are highly conserved in rhodopsin-like G protein-coupled receptors (Moro et al., 1993; Eilers et al., 2005). The conserved amino acid residues that are responsible for coupling the mAChRs to Gq/11 (Ren et al., 2015) are found in the IL2 and IL3 of *M. separata* A-type mAChR (marked with a triangle in Figure 1). The motif WXFG in extracellular loop one (EL1) is highly conserved across Class A GPCRs, which plays a critical role in GPCR targeting to the plasma membrane (Rizzo et al., 2018). The Ms A-type mAChR shares identity to the orthologs in *T. castaneum* (EFA01319), *Drosophila* (AFJ23965), and *Apis mellifera* (XP_395760) is 65, 64, and 61%, respectively, and shares 37% identity to B-type and 26% to C-type receptors of *Drosophila*. Phylogenetic analysis of all insect mAChRs and human m1–m5 mAChRs revealed that the insect A-type mAChRs grouped together and clustered with human m1–m5 mAChRs, and then clustered with insect B-type mAChRs (Figure 2). Interestingly, the C-type receptors divided from the mAChRs and formed a different cluster. The Ms A-type mAChR was most closely related to *Drosophila* A-mAChR (Figure 2).

### Functional and Pharmacological Characterization of Ms A-Type mAChR

**FIGURE 3:**
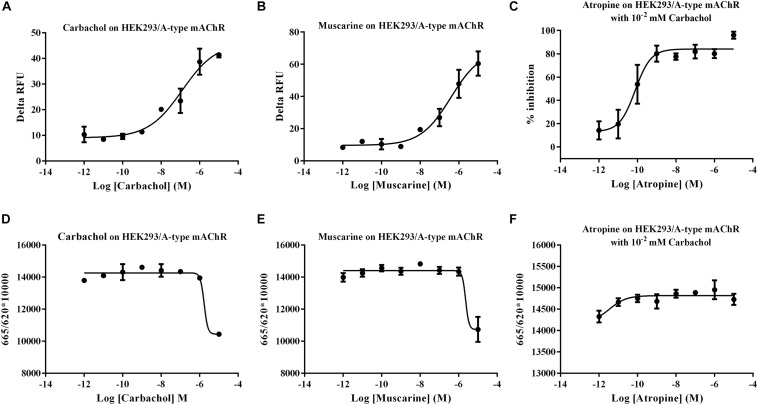
Functional assay of *Mythimna separata* A-type mAChR transiently expressed in HEK293 cell lines. **(A)** Dose–response curves of the effects of carbachol on representative [Ca^2+^] i responses. **(B)** Dose–response curves of the effects of muscarine on representative [Ca^2+^] i responses. **(C)** Dose–response curves of the effects of antagonist atropine on the [Ca^2+^] i responses induced by 10 μM carbachol. **(D)** Dose–response curves of the effects of carbachol on modulation of intracellular cAMP of A-type mAChR transiently expressed in HEK293 cell lines. **(E)** Dose–response curves of the effects of muscarine on modulation of intracellular cAMP of A-type mAChR transiently expressed in HEK293 cell lines. **(F)** Dose–response curves of the effects of antagonist atropine on intracellular cAMP responses induced by 10 μM carbachol.

Human embryonic kidney (HEK) 293 cells transiently expressing the putative Ms A-type mAChR was used to functionally characterize the pharmacological properties of this gene. Ligand–receptor interaction was monitored using a calcium mobilization assay and a cAMP assay. The result showed that a low concentration (10 nM) of carbachol and muscarine can apparently induce the increase of the intracellular Ca^2+^ level, and the Ms A-mAChR was robustly activated by carbachol (Figure 3A) and muscarine (Figure 3B) in a dose-dependent manner, with the EC_50_ (half maximal effective concentration) value of 124.6 and 388.1 nM. These agonists did not show any Ca^2+^ response in untransfected HEK-293 cells or GFP transfected HEK293 cells. For assays with an antagonist, the elevation of the Ca^2+^ level caused by 10 μM carbachol was significantly reduced by the classical antagonist atropine (80% inhibition at 1 nM), with an IC_50_ (half maximal inhibition concentration) value of 0.09 nM (Figure 3C). For cAMP assay, it was strange that 10 μM carbachol (Figure 3D) and muscarine (Figure 3E) can increase the concentration of intracellular cAMP, but no response was found with a concentration of agonist of less than 1 μM (Figures 3D,E). The control cells (GFP transfected HEK293 cells or untransfected cells) did not show a cAMP response to carbachol or muscarine (data not shown). And for assays with a dilution series of antagonist, no apparent increase of cAMP was detected in the cells treated with 10 μM carbachol (Figure 3F). The substitution of 0.1% DMSO for the agonist was used as another control, and no response was observed in the Ms A-mAChR transfected cells (data not shown).

### Expression Profiles of A-Type mAChR mRNA in *M. separata*

**FIGURE 4:**
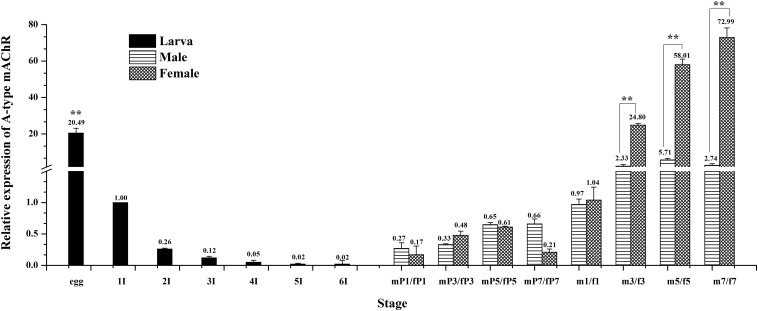
Relative expression profiles (±standard error of the mean; *n* = 3) of Ms A-type mAChR mRNA in different developmental stages of *Mythimna separata*. All expression levels are shown relative to the expression level in the first instar. Developmental stages from egg to the last instar; **highly significant difference [*P* ≦ 0.01, analysis of variance (ANOVA)] compared with other larval stages. Developmental stages from pupae to adults of male and female *M. separata*. **highly significant difference [*P* ≦ 0.01, analysis of variance (ANOVA)] compared with males of the same developmental stage.

The expression levels of Ms A-type mAChR transcripts in different developmental stages of *M. separata* were detected by qRT-PCR. Results show that it is extensively expressed in all developmental stages. The lowest expression level is found in larvae stages and the highest in adults (Figure 4). During larval development, the transcripts are highest in the egg (3 days after laying) and decline greatly in the first instar (only one-third of amount in eggs), then gradually decreasing with further development and is lowest in the sixth instar before pupation (Figure 4). In the pupal stage, it becomes possible to distinguish between males and females. Expression levels increase slightly but have not reached the level of the first instar, maintaining at a low level in both male and female pupae of different developmental stages, and with no apparent difference in expression between male and female pupae (Figure 4). But the expression levels increase greatly in adult stage, at the first day of eclosion, just reaching to that of the first instar, and with no apparent difference between male and female adults 1 day after eclosion. However, the expression level increases greatly at the third day of eclosion, with nearly 24 times more in females than that in the first instar. Furthermore, there is a great difference between male and females, with 12 times more in females than that in males. This apparent differential expression level between male and female adults was maintained through the following days, reached to 26 times more in females than that in males at 7 days after eclosion (Figure 4).

**FIGURE 5:**
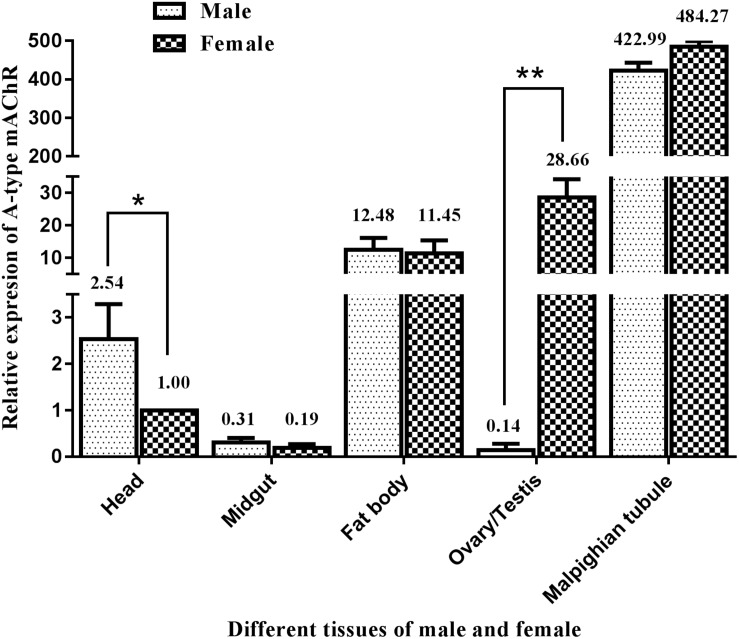
Relative expression profiles (±standard error of the mean; *n* = 3) of Ms A-type mAChR mRNA in different tissues of *Mythimna separata* adults. All expression levels are shown relative to the expression level in the head of female adults. **highly significant difference [*P*≦0.01, analysis of variance (ANOVA)], *significant difference (*P* ≦ 0.05, ANOVA).

Similarly, qRT-PCR was performed to determine the differential expression of A-type mAChR mRNA in different tissues of the male and female adults at 3 days after eclosion. The results show that there is also a widespread distribution of A-type mAChR in the various tissues of *M. separata*. Unexpectedly, the highest expression was not in the head but in the Malpighian tubules in both the males and females, followed by ovary, fat body, and head. The lowest expression was found in the midgut and testis (Figure 5). Using the heads of females for calibration, the gene transcription level was found to be 484-fold higher in the Malpighian tubules of females and 423-fold higher in that of males compared with the head calibration designated as 1. Surprisingly, a greatly significant differential expression between males and females was found in the reproductive system, the expression level in the ovary being more than 204 times higher than in the testis (28.66 in ovary and 0.14 in testis). There were also measurable differences in receptor levels of the head between males and females (Figure 5).

To verify whether there exists different Ms A-type mAChR isoforms between the brains and other tissues (ovaries and Malpighian tubules), the receptor sequence was cloned by using gene specific primers (S2, A2) across ORF from the brain, ovary and Malpighian tubules cDNAs, respectively. The result showed that there was no difference between the receptor sequences from different tissues.

### Ms A-mAChR Protein Distribution

**FIGURE 6:**
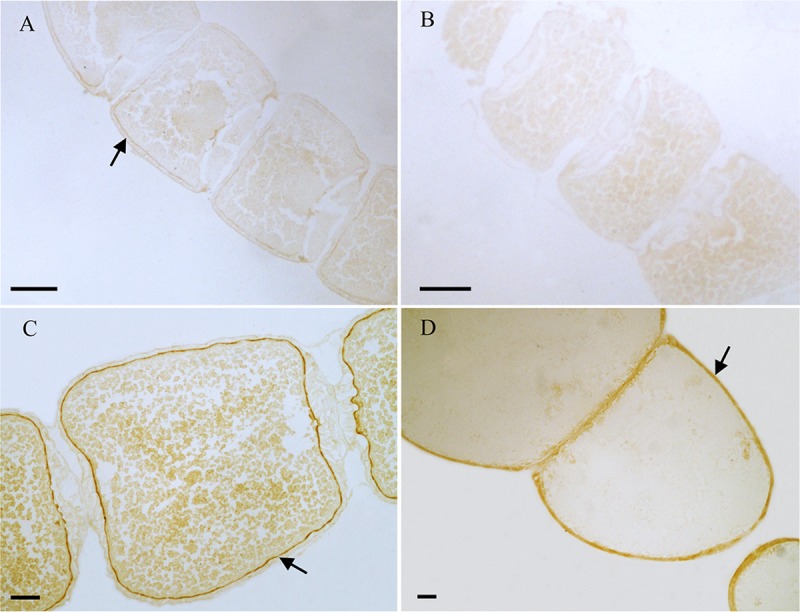
Immunostaining in the ovary of female *Mythimna separata* adults with polyclonal antiserum against Ms A-type mAChR protein. Streptavidin-peroxidase (SP) staining technique, cryosections. **(A)** Arrows showing the moderate positive signal at the membrane of the oocyte in the early stage of oogenesis. **(B)** The negative control, showing no positive signals. **(C,D)** Arrows the strong positive signal at the membrane of the oocytes and the old development oocytes. Scale bar = 200 μm.

**FIGURE 7:**
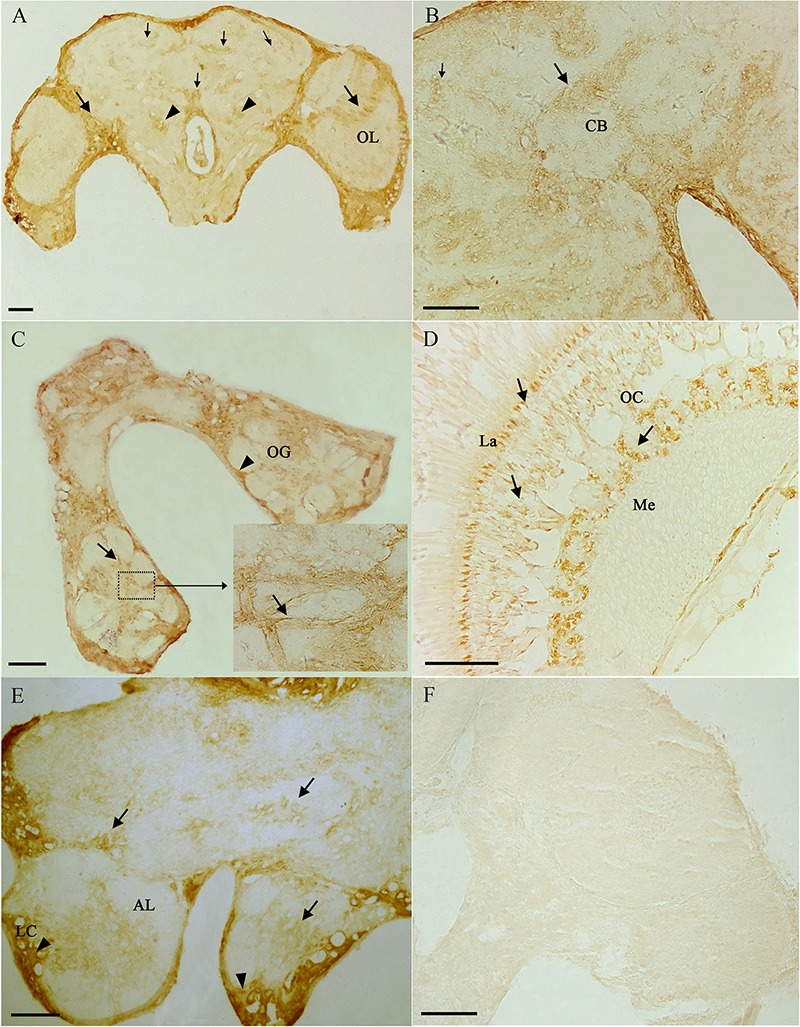
Immunostaining in the brain of male *Mythimna separata* adults with polyclonal antiserum against Ms A-type mAChR protein. streptavidin-peroxidase (SP) staining technique, cryosections. **(A)** Frontal section of the brain of males showing the distribution pattern of Ms A-type mAChR protein. Large arrows showing Ms A-type mAChR immunostaining in cell clusters and fibers of the optic lobe (OL), arrowheads showing Ms A-type mAChR immunostaining in antennal lobes (ALs), small arrow showing Ms A-type mAChR immunostaining in the central body (CB) and mushroom body (MB). **(B)** Arrows showing Ms A-type mAChR immunostaining in CB; small arrow showing Ms A-type mAChR immunostaining in MB. **(C)** Arrows showing Ms A-type mAChR immunostaining in the ordinary glomeruli (OG) and fibrous core of ALs. Arrowheads showing Ms A-type mAChR immunostaining in the lateral cell cluster (LC) of AL. **(D)** Frontal section through the OL showing Ms A-type mAChR immunostaining in the proximal layer of the lamina (La), fibers in the optic chiasm (OC) and the medulla (Me). **(E)** Arrowheads showing Ms A-type mAChR immunostaining in the LC of AL and in the CB, arrows showing Ms A-type mAChR immunostaining in OG of AL. **(F)** The negative control, showing no positive signals. Scale bar = 200 μm.

From the results of tissues mRNA distribution of Ms A-mAChR, we are interested in its distribution in the ovary of female as well as the brain of males. A polyclonal antiserum raised against a synthetic peptide corresponding to the C terminus of Ms A-mAChR was used to determine the distribution of the protein. The results show that during oogenesis, Ms A-mAChR protein was detected on the membrane of different developmental stages of oocyte. No positive signal was found in the nutrient cells or the follicular cells (Figure 6). During the early stages of oogenesis, there was a moderate positive signal at the membrane of the oocyte (Figure 6A). As the oocyte grows, this positive reaction became stronger and was also mainly distributed at the membrane of the oocyte (Figure 6C) and fully grown oocytes (Figure 6D). There was no positive signal in the controls (Figure 6B). In the brain of the male adults, Ms A-mAChR protein was mainly distributed in the antennal lobes (ALs), the optic lobes (OLs), and moderate expression was found in mushroom bodies (MBs) and the central complex of the brain (Figure 7). In the ALs, Ms A-mAChR protein was located along the fibers of ordinary glomeruli and in the lateral cell cluster (Figures 7C,E). The protein was also found in the proximal layer of the lamina (Figure 7D), fibers in the optic chiasm and the medulla of OLs near the optic chiasm (Figure 7D) as well as the fibers in OLs (Figure 7A). There was no positive signal in the control (Figure 7F).

## Discussion

### Molecular Properties of the Ms A-Type mAChR

The full-length cDNA sequence of an A-type mAChR from *M. separata* was identified by degenerative PCR and Race methods. The presence of seven potential membrane-spanning segments and the G-protein coupled receptors signature (Figure 1) revealed that it was a GPCR. When blasted in the protein database, it shares a high degree of sequence similarity (>50%) with the A-type mAChRs of insects. We therefore designated it as Ms A-type mAChR. The phylogenetic analysis including insect A-/B-/C-type mAChRs and human m1–m5 receptors show that Ms A-type mAChR is mostly related to *Drosophila* A-type mAChR. The insect A-type mAChRs group together and cluster closely with human m1–m5 receptors, but not insect B- or C-type receptors (Figure 2). This indicates that the insect A-type receptors may be structurally similar to human mAChRs or have similar pharmacological properties to human mAChRs. Further functional motifs analysis revealed that the amino acid residues responsible for coupling the mAChRs to G_*q/*__11_ (Ren et al., 2015) and the amino acid residues involved in the antagonist QNB binding (Fryer et al., 2012) are also found in Ms A-type mAChR. It appears that all mAChRs known to be coupled to G_*q/*__11_ have these identical amino acid residues in common, which are absent in the mAChRs known to be coupled to G_*i/*__0_ (Ren et al., 2015). Thus, we speculate that Ms A-type mAChR may couple to G_*q/*__11_ to activate phospholipase C. The six amino acid residues (Figure 1) that are responsible for ligand binding (Haga et al., 2011) are also found in the Ms A-mAChR, they are the same as in other insect and human mAChRs (Ren et al., 2015), which is consistent with the fact that ACh is the natural agonist, having comparable efficacy for these receptors (Collin et al., 2013).

### Functional Coupling to Intracellular Second Messenger Pathways

HEK293 cells are widely used as a cell-based model for the transfection of various mAChRs including the vertebrate and invertebrate mAChRs (Lee et al., 1998; von der Kammer et al., 1998; Thomas and Smart, 2005; Alfa Cissé et al., 2007; Luo et al., 2008) to investigate cell signaling pathway. In order to characterize the signal properties of Ms A-type mAChR, cDNA encoding the receptor was transiently transfected into HEK 293 cells, which have also been used successfully in previous studies to examine the pharmacological properties of cloned insect GPCRs (Blenau et al., 1998; Grohmann et al., 2003; Huang et al., 2009). Activation of heterologously expressed Ms A-type mAChR by agonists carbachol and muscarine led to a great increase of intracellular Ca^2+^concentration (Figure 3). It is more sensitive to carbachol than muscarine. And nanomolar concentrations of the ligand are sufficient to evoke the Ca^2+^ signal. The classical antagonist atropine, a competitive muscarinic antagonist (Smith et al., 1994), can significantly reduce the elevation of Ca^2+^ level caused by 10 μM carbachol. Previous research revealed that the insect A-type mAChRs can be activated by low concentrations (10^–8^ M) of ACh and muscarine, and blocked by the classical mAChR antagonists atropine, scopolamine, and QNB (Collin et al., 2013). The pharmacological profile of Ms A-type mAChR receptor resembles that of the *Drosophila* and other insect A-type mAChRs (Shapiro et al., 1989; Collin et al., 2013). The vertebrate m2 and m4 receptors preferentially coupled to members of the G_*i/*__0_ family, leading to a decrease of intracellular cAMP (Shapiro et al., 1988; Eglen, 2005; Giglio and Tobin, 2009; Bubser et al., 2012). In insect, a presynaptic m2 subtype, which inhibits cAMP synthesis, was detected in locust ganglia (Knipper and Breer, 1988). In order to determine if the cloned receptor is able to inhibit adenylate cyclase activity when expressed in HEK293 cells, the concentration of intracellular cAMP was detected with various concentrations of agonists and antagonist. Interestingly, no decrease of intracellular cAMP concentration was observed in response to the agonists. But transfected cells displayed an increase of cAMP concentration when high concentrations of carbachol or muscarine (10 μM) are applied (Figures 3D,E). This phenomenon was also found in Shapiro’s research about *Drosophila* mAChR (Shapiro et al., 1989). When *Drosophila* mAChR was expressed in mouse Y1 adrenal cells, the agonist carbachol stimulated the production of inositol phosphates. The carbachol also increased the production of cAMP. They speculated that one of the limitations of this approach (shared with the expression studies on vertebrate mAChRs) is that the coupling of foreign receptors can depend both on the host cells and on the receptor densities (Trimmer, 1995). Moreover, Grohmann’s research about an octopamine receptor (Grohmann et al., 2003) found that nanomolar to micromolar concentrations of octopamine induced the increase of the intracellular Ca^2+^concentration, but incubation of Amoa1-transfected HEK 293 cells with high concentration of octopamine (10 μM) would also cause a significantly higher level of cAMP production. They supposed that the increase in intracellular cAMP concentration observed at high octopamine concentration was most probably a secondary effect, induced by massive Ca^2+^ release. Previous research also indicated that heterologously expressed GPCRs can activate different intracellular signaling systems, depending on the cell line used for expression and the agonist used for receptor stimulation (Robb et al., 1994; Reale et al., 1997; Sidhu and Niznik, 2000). In the case of Ms A-type mAChR, activation of heterologously expressed Ms A-type mAChR with agonists at physiological concentrations specifically causes the increase of Ca^2+^ concentration. These findings are consistent with the bioinformatics analysis that Ms A-type mAChR owned the amino acid residues responsible for coupling to G_*q/*__11_, and clustered with insect A-type mAChR. The cAMP response, however, is only observed when high concentrations of agonists (10 μM) are applied. The control cells (GFP transfected HEK293 cells or untransfected cells) did not show any cAMP response to carbachol or muscarine even with high concentration (10 μM). Antagonist atropine even with low concentration would suppress this effect. Therefore, we speculated that the Ms A-type mAChRs couple to G_*q/*__11_ to activate phospholipase C, leading to a signaling cascade that produces an increase in cellular Ca^2+^ concentration. The massive increase in [Ca^2+^]_*i*_ caused by stimulation with high concentrations of agonists may activate the adenylyl cyclase in a secondary reaction. However, further research would perform to reveal this speculation.

### Functional Implications of the Ms A-Type mAChR

Previous research revealed that AChRs in insects appear to be expressed exclusively in the nervous system (Sattelle, 1980; Breer and Sattelle, 1987). More attention was focused on its physiological function within the nervous system through the application of muscarine agonists (Hoffmann et al., 2007; Heck et al., 2009). In this study, we found that a basal Ms A-type mAChR transcription was detected in all developmental stages (Figure 4), indicating the consistent expression of Ms A-type mAChR throughout *M. separata* development. However, expression level was higher in egg and adult stages, and especially in female adults. The transcript level was approximately 73-fold higher in female adults at 7 days after eclosion, relative to the first instar. Lower levels were always found in the larval and pupal stages. These results may suggest an important biological role for Ms A-type mAChR in female adults. A-type mAChR expression analysis in *D. melanogaster* revealed that a high expression level is found in 3-day-old male and female pupae and in male adults, while female adults contain about 4–12 times lower concentrations of the mRNAs for the receptor compared to the males (Collin et al., 2013). In our result, Ms A-type mAChR mRNAs maintain a constant lower level across all pupal developmental stages, while female adults contain about 10–26 times higher concentrations of A-type mAChR transcripts than the male adults. This may indicate that the physiological roles of A-type mAChR are different between Diptera and Lepidoptera.

In order to determine where the high concentration of Ms A-type mAChR mRNA is distributed in adults, we identified the tissue expression profiles in male and female adults at 3 days after eclosion. Surprisingly, the highest expression was not found in the head, but in the Malpighian tubules of both males and females. This result is different from *D. melanogaster*, where both A- and B-type mAChRs were found mainly in the head compared to the thorax and abdomen (Collin et al., 2013). Furthermore, there was 2.54 times higher level of Ms A-type mAChR transcripts in the male head compared to the female head. Further immunohistochemistry study found that the Ms A-type mAChR protein mainly located in the ALs, the OLs, the MBs, and the central complex of the male brain. This general result is similar to the mRNA distribution patterns previously described for mAChR from the ant *P. vicina* (Lü et al., 2011) and the mAChR protein distribution in *Drosophila* (Blake et al., 1993). It further establishes the neuroactive roles of Ms A-type mAChR in the brain of *M. separata*. As we known, ALs and OLs are the primary integration center for olfactory and optical information, respectively. And the MBs receive afferents from the primary sensory regions of the brain (Gronenberg, 2001; Farris and Sinakevitch, 2003). In addition, the connection between the AL projection neurons and the MB calyces is cholinergic (Hansson and Anton, 2000; Lozano et al., 2001), the cholinergic signaling via muscarinic receptors plays a role in olfaction-based social behavior in honey bees (Ismail et al., 2008). Moreover, in *Drosophila* and honeybee, pharmacological studies revealed that mAChRs take great roles in the formation and recall of memory, and couple experience to structural brain plasticity (Lozano et al., 2001; Ismail et al., 2006; Silva et al., 2015). In this study, the high expression of Ms A-type mAChR in the ALs and the OLs may suggest the function of this receptor in integrating the primary olfactory and visual information. The MBs are responsible for higher order integration of sensory information and involved in multimodal sensory integration and certain forms of learning and memory. The moderate expression of Ms A-type mAChR in the MBs may indicate this receptor is also involved in high-order integration of olfactory and optical information. It is consistent with the pharmacological studies of mAchRs in the honeybee (Lozano and Gauthier, 1998; Lozano et al., 2001; Ismail et al., 2008). Taken together, we can propose that Ms A-type mAChR in the brain may be related to its neural physiological function in males for modulating the olfactory and visual information.

In vertebrates, epithelial ACh (non-neuronal) has been shown to be released into the lining fluid and is involved in the regulation of ion- and water transport (Hollenhorst et al., 2012). From our results on signaling properties of this receptor, the Ms A-type mAChR coupled to G_*q/*__11_ and induced the increase of intracellular Ca^2+^ concentration. It seems that the highest expression of Ms A-type mAChR in Malpighian tubules may be related to its excretory function, for example, the transport of ions and water. This speculation needs to be further identified.

We are more interested in the greatly differential expression in the reproductive system of males and females (204 times more in ovary than in testis). Previous research revealed that circular and longitudinal muscles were detected in the ovaries of a number of Lepidoptera, including the flour moth *Ephestia kühniella* (Cruickshank, 1973), the sugar cane borer *Diatraea saccharalis* (dos Santos and Gregório, 2002), and the butterfly *Calpodes ethlius* (Griffith and Lai-Fook, 1986). We therefore speculate that the high expression of Ms A-type mAChR in ovary may be related to the movement of the eggs down the tract from ovarioles. Surprisingly, Ms A-mAChR protein is localized on the membrane of different developmental stages of oocytes that are not innervated by neurons at all. And no Ms A-mAChR protein was found in the nutrient cells or the follicular cells. This membrane-bound pattern corresponds with the fact that mAChR is a membrane receptor.

In vertebrates, ACh and the pivotal components of the cholinergic system are expressed by the majority of cells not innervated by neurons at all, which are vital for various types of cells such as epithelial, endothelial and immune cells (Wessler and Kirkpatrick, 2008; Kummer and Krastevachrist, 2014). m3 and m4 receptors were also found in mouse oocytes to be linked to Ca^2+^ oscillations (Kang et al., 2003). And oocytes of some vertebrate species have been found to synthesize ACh and express muscarinic receptors (Eusebi et al., 1984; Fritz et al., 2001; Angelini et al., 2004). Honeybees can produce millimolar concentrations of non-neuronal ACh for breeding and the fertilized oocytes also contain ACh (Wessler et al., 2016). In the sphinx moth *M. sexta*, the muscarinic cholinergic system was not restricted to the sensory neurons but was also present in glial and epidermal cells (Clark et al., 2005), and muscarinic cholinergic interactions between the neural and non-neural cells during the development of *Manduca antenna* (Torkkeli et al., 2005). However, our knowledge about a possible role of ACh and the cholinergic system in reproduction processes of non-mammalian animals is very scanty (Wessler and Kirkpatrick, 2017). In our result, the high level of Ms A-mAChR protein located on the membrane of oocytes which are completely devoid of any innervation may suggest a new non-neuronal function of A-type mAChR in the female reproductive system that has never been reported for insects. Non-neuronal cholinergic systems have been introduced to describe the expression and biological role of ACh released from non-neuronal cells to communicate with neighboring non-neuronal cells and also to trigger intracellular signaling independent of neuronal input (Wessler et al., 1998, 1999). We are interested in where the ACh in ovaries of the armyworm comes from. If it is released from ovarian innervation, ACh would have to reach this cellular compartment by diffusion. However, ACh is a labile and short-lived molecule (Mayerhofer and Kunz, 2005). Further experiments to determine where the ACh comes from and pivotal components of the cholinergic system in ovary of *M. separata* are in progress.

## Data Availability Statement

The datasets generated for this study can be found in the GenBank accession no. KY296116.

## Author Contributions

SL, MJ, XT, SH, and JZ carried out the experiments. SL and MJ performed the statistical analysis. SL and YZ designed the study and wrote the manuscript. All authors agreed to be accountable for the content of the work.

## Conflict of Interest

The authors declare that the research was conducted in the absence of any commercial or financial relationships that could be construed as a potential conflict of interest.

## Footnotes

1 www.ncbi.nlm.nih.gov

2 http://www.expasy.org/

3 http://www.cbs.dtu.dk/services/TMHMM/

4 http://www.predictprotein.org/

5 http://www.ebi.ac.uk/Tools/msa/clustalw

6 http://www.megasoftware.net

7 http://www.genscript.com/antigen-design.html

8 http://www.ncbi.nlm.nih.gov/blast/Blast.cgi

## References

[B1] Aizono Y. Endo Y. Sattelle D. B. Shirai Y. (1997). Prothoracicotropic hormone-producing neurosecretory cells in the silkworm. *Bombyx mori*, express a muscarinic acetylcholine receptor. *Brain Res.* 763 131–136. 10.1016/s0006-8993(97)00496-4 9272838

[B2] Alfa Cissé M. Sunyach C. Slack B. E. Fisher A. Vincent B. Checler F. (2007). M1 and M3 muscarinic receptors control physiological processing of cellular prion by modulating ADAM17 phosphorylation and activity. *J. Neurosci.* 27 4083–4092. 10.1523/jneurosci.5293-06.2007 17428986 PMC6672535

[B3] Altschul S. F. Madden T. L. Schäffer A. A. Zhang J. Zhang Z. Miller W. (1997). Gapped BLAST and PSI-BLAST: a new generation of protein database search programs. *Nucleic Acids Res.* 25 3389–3402. 10.1093/nar/25.17.3389 9254694 PMC146917

[B4] Angelini C. Baccetti B. Piomboni P. Trombino S. Aluigi M. G. Stringara S. (2004). Acetylcholine synthesis and possible functions during sea urchin development. *Eur. J. Histochem.* 48 235–243. 15590413

[B5] Blake A. D. Anthony N. M. Chen H. H. Harrison J. B. Nathanson N. M. Sattelle D. B. (1993). *Drosophila* nervous system muscarinic acetylcholine receptor: transient functional expression and localization by immunocytochemistry. *Mol. Pharmacol.* 44 716–724. 8232221

[B6] Blenau W. Erber J. Baumann A. (1998). Characterization of a dopamine D1 receptor from *Apis mellifera*: cloning, functional expression, pharmacology, and mRNA localization in the brain. *J. Neurochem.* 70 15–23. 10.1046/j.1471-4159.1998.70010015.x 9422342

[B7] Breer H. Sattelle D. B. (1987). Molecular properties and functions of insect acetylcholine receptors. *J. Insect. Physiol.* 33 771–790. 10.1016/0022-1910(87)90025-4

[B8] Brody T. Cravchik A. (2000). *Drosophila melanogaster* G protein-coupled receptors. *J. Cell. Biol.* 150 83–88. 10.1083/jcb.150.2.f83 PMC2180217 10908591

[B9] Bubser M. Byun N. Wood M. R. Jones C. K. (2012). Muscarinic receptor pharmacology and circuitry for the modulation of cognition. *Handb. Exp. Pharmacol.* 208 121–166. 10.1007/978-3-642-23274-9_7 22222698

[B10] Casida J. E. (2018). Neonicotinoids and other insect nicotinic receptor competitive modulators: progress and prospects. *Annu. Rev. Entomol.* 63 125–144. 10.1146/annurev-ento-020117-043042 29324040

[B11] Caulfield M. P. (1993). Muscarinic Receptors—Characterization, coupling and function. *Pharmacol. Ther.* 58 319–379. 10.1016/0163-7258(93)90027-b 7504306

[B12] Chen C. Okayama H. (1987). High-efficiency transformation of mammalian cells by plasmid DNA. *Mol. Cell Biol.* 7 2745–2752. 10.1128/mcb.7.8.2745 3670292 PMC367891

[B13] Clark J. Meisner S. Torkkeli P. H. (2005). Immunocytochemical localization of choline acetyltransferase and muscarinic ACh receptors in the antenna during development of the sphinx moth *Manduca sexta*. *Cell Tissue Res.* 320 163–173. 10.1007/s00441-004-1039-7 15719247

[B14] Collin C. Hauser F. Gonzalez de Valdivia E. Li S. Reisenberger J. Carlsen E. M. M. (2013). Two types of muscarinic acetylcholine receptors in *Drosophila* and other arthropods. *Cell. Mol. Life Sci.* 70 3231–3242. 10.1007/s00018-013-1334-0 23604020 PMC11113683

[B15] Cruickshank W. J. (1973). The ultrastructure and functions of the ovariole sheath and tunica propria in the flour moth. *J. Insect. Physiol.* 19 577–592. 10.1016/0022-1910(73)90067-x

[B16] Dick M. R. Dripps J. E. Orr N. (1997). Muscarinic agonists as insecticides and acaricides. *Pestic. Sci.* 49 268–276. 10.1002/(sici)1096-9063(199703)49:3<268::aid-ps527>3.0.co;2-c

[B17] dos Santos D. C. Gregório E. A. (2002). Ultrastructure of the ovariole sheath in *Diatraea saccharalis* (Lepidoptera: Pyralidae). *Biocell* 26 229–235. 12240557

[B18] Dupuis J. Louis T. Gauthier M. Raymond V. (2012). Insights from honeybee (*Apis mellifera*) and fly (*Drosophila melanogaster*) nicotinic acetylcholine receptors: from genes to behavioral functions. *Neurosci. Biobehav. Rev.* 36 1553–1564. 10.1016/j.neubiorev.2012.04.003 22525891

[B19] Eglen R. M. (2005). Muscarinic receptor subtype pharmacology and physiology. *Prog. Med. Chem.* 43 105–136. 10.1016/s0079-6468(05)43004-0 15850824

[B20] Eilers M. Hornak V. Smith S. O. Konopka J. B. (2005). Comparison of class A and D G protein-coupled receptors: common features in structure and activation. *Biochemistry* 44 8959–8975. 10.1021/bi047316u 15966721 PMC1382269

[B21] Eusebi F. Pasetto N. Siracusa G. (1984). Acetylcholine receptors in human oocytes. *J. Physiol.* 346 321–330. 10.1113/jphysiol.1984.sp015024 6699777 PMC1199501

[B22] Farris S. M. Sinakevitch I. (2003). Development and evolution of the insect mushroom bodies: towards the understanding of conserved developmental mechanisms in a higher brain center. *Arthropod. Struct. Dev.* 32 79–101. 10.1016/s1467-8039(03)00009-4 18088997

[B23] Feng H. Q. Zhao X. C. Wu X. F. Wu B. Wu K. M. Cheng D. F. (2008). Autumn migration of *Mythimna separata* (Lepidoptera: Noctuidae) over the Bohai Sea in Northern China. *Environ. Entomol.* 37 774–781. 10.1603/0046-225x(2008)37[774:amomsl]2.0.co;2 18559184

[B24] Fritz S. Wessler I. Breitling R. Rossmanith W. Ojeda S. R. Dissen G. A. (2001). Expression of muscarinic receptor types in the primate ovary and evidence for nonneuronal acetylcholine synthesis. *J. Clin. Endocrinol. Metab.* 86 349–354. 10.1210/jc.86.1.349 11232023

[B25] Fryer A. D. Christopoulos A. Nathanson N. M. (2012). *Muscarinic Receptors.* Cham: Springer, 359–360.

[B26] Gauthier M. Cano Lozano V. Zaoujal A. Richard D. (1994). Effects of intracranial injections of scopolamine on olfactory conditioning in the honeybee. *Behav. Brain Res.* 63 145–149. 10.1016/0166-4328(94)90085-x 7999297

[B27] Giglio D. Tobin G. (2009). Muscarinic receptor subtypes in the lower urinary tract. *Pharmacology* 60 3–21. 10.1159/000209255 19295256

[B28] Gorczyca M. G. Budnik V. White K. Wu C. F. (1991). Dual muscarinic and nicotinic action on a motor program in *Drosophila*. *J. Neurobiol.* 22 391–404. 10.1002/neu.480220407 1679841

[B29] Greenspan R. J. (1980). Mutations of choline acetyltransferase and associated neural defects. *J. Comp. Physiol. A.* 137 83–92. 10.1007/bf00656920

[B30] Greenspan R. J. Finn J. A. Jr. Hall J. C. (1980). Acetylcholinesterase mutants in *Drosophila* and their effects on the structure and function of the central nervous system. *J. Comp. Neurol.* 189 741–774. 10.1002/cne.901890409 6769980

[B31] Griffith C. M. Lai-Fook J. (1986). The ovaries and changes in their structural components at the end of vitellogenesis and during vitelline membrane formation in the butterfly, *Calpodes*. *Tissue Cell* 18 575–588. 10.1016/0040-8166(86)90022-4 18620173

[B32] Grohmann L. Blenau W. Erber J. Ebert P. R. Strünker T. Baumann A. (2003). Molecular and functional characterization of an octopamine receptor from honeybee (*Apis mellifera*) brain. *J. Neurochem.* 86:725. 10.1046/j.1471-4159.2003.01876.x 12859685

[B33] Gronenberg W. (2001). Subdivisions of hymenopteran mushroom body calyces by their afferent supply. *J. Comp. Neurol.* 436 474–489. 10.1002/cne.1045 11406827

[B34] Gross A. D. Bloomquist J. R. (2018). Pharmacology of central octopaminergic and muscarinic pathways in *Drosophila melanogaster* larvae: assessing the target potential of GPCRs. *Pestic. Biochem. Physiol.* 151 53–58. 10.1016/j.pestbp.2018.08.001 30704713

[B35] Haga K. Kruse A. C. Asada H. Yurugi-Kobayashi T. Shiroishi M. Zhang C. (2011). Structure of the human M2 muscarinic acetylcholine receptor bound to an antagonist. *Nature* 482 547–551. 10.1038/nature10753 22278061 PMC3345277

[B36] Hansson B. S. Anton S. (2000). Function and morphology of the antennal lobe: new developments. *Annu. Rev. Entomol.* 45 203–231. 10.1146/annurev.ento.45.1.203 10761576

[B37] Harrison J. B. Chen H. H. Blake A. D. Huskisstm N. S. Baker P. Sattelle D. B. (1995). Localization in the nervous system of *Drosophila melanogaster* of a C-terminus anti-peptide antibody to a cloned *Drosophiha* muscarinic acetylcholine receptor. *J. Neuroendocrinol*. 7 347–352. 10.1111/j.1365-2826.1995.tb00768.x 7550280

[B38] Heck C. Kunst M. Härtel K. Hülsmann S. Heinrich R. (2009). In vivo labeling and in vitro characterisation of central complex neurons involved in the control of sound production. *J. Neurosci. Methods* 183 202–212. 10.1016/j.jneumeth.2009.06.032 19583981

[B39] Hoffmann K. Wirmer A. Kunst M. Gocht D. Heinrich R. (2007). Muscarinic excitation in grasshopper song control circuits is limited by acetylcholinesterase activity. *Zool. Sci.* 24:1028. 10.2108/zsj.24.1028 18088166

[B40] Hollenhorst M. I. Lips K. S. Wolff M. Wess J. Gerbig S. Takats Z. (2012). Luminal cholinergic signalling in airway lining fluid: a novel mechanism for activating chloride secretion via Ca 2+ -dependent Cl - and K + channels. *Br. J. Pharmacol.* 166:1388. 10.1111/j.1476-5381.2012.01883.x 22300281 PMC3417454

[B41] Honda H. Tomizawa M. Casida J. E. (2007). Insect muscarinic acetylcholine receptor: pharmacological and toxicological profiles of antagonists and agonists. *J. Agric. Food Chem.* 55 2276–2281. 10.1021/jf0631934 17319687

[B42] Huang J. Ohta H. Inoue N. Takao H. Kita T. Ozoe F. (2009). Molecular cloning and pharmacological characterization of a *Bombyx mori* tyramine receptor selectively coupled to intracellular calcium mobilization. *Insect. Biochem. Mol. Biol.* 39 842–849. 10.1016/j.ibmb.2009.10.001 19833207

[B43] Hue B. Lapied B. Malecot C. O. (1989). Do presynaptic rnuscarinic receptors regulate acetylcholine release in the central nervous system of the cockroach *Prripluizefu umericana*? *J. Exp. Biol.* 142:447.

[B44] Ihara M. Buckingham S. D. Matsuda K. Sattelle D. B. (2017). Modes of action, resistance and toxicity of insecticides targeting nicotinic acetylcholine receptors. *Curr. Med. Chem.* 24 2925–2934. 10.2174/0929867324666170206142019 28176635

[B45] Ismail N. Christine S. Robinson G. E. Fahrbach S. E. (2008). Pilocarpine improves recognition of nestmates in young honey bees. *Neurosci. Lett.* 439 178–181. 10.1016/j.neulet.2008.05.014 18514413 PMC2517128

[B46] Ismail N. Robinson G. E. Fahrbach S. E. (2006). Stimulation of muscarinic receptors mimics experience-dependent plasticity in the honey bee brain. *Proc. Natl. Acad. Sci. U.S.A.* 103 207–211. 10.1073/pnas.0508318102 16373504 PMC1324993

[B47] Jones A. K. Sattelle D. B. (2010). Diversity of insect nicotinic acetylcholine receptor subunits. *Adv. Exp. Med. Biol.* 683 25–43. 10.1007/978-1-4419-6445-8_3 20737786

[B48] Kang D. Park J. Y. Han J. Bae I. H. Yoon S. Y. Kang S. S. (2003). Acetylcholine induces Ca2+ oscillations via m3/m4 muscarinic receptors in the mouse oocyte. *Pflugers Arch.* 447 321–327. 10.1007/s00424-003-1184-y 14557882

[B49] Knipper M. Breer H. (1988). Subtypes of muscarinic receptors in insect nervous system. *Comp. Biochem. Physiol.* 90 275–280. 10.1016/0742-8413(88)90133-8

[B50] Kummer W. Krastevachrist G. (2014). Non-neuronal cholinergic airway epithelium biology. *Curr. Opin. Pharmacol.* 16 43–49. 10.1016/j.coph.2014.03.001 24681350

[B51] Lee K. B. Pals-Rylaarsdam R. Benovic J. L. Hosey M. M. (1998). Arrestin-independent internalization of the m1, m3, and m4 subtypes of muscarinic cholinergic receptors. *J. Biol. Chem.* 273 12967–12972. 10.1074/jbc.273.21.12967 9582330

[B52] Li K. Yin H. Xi G. S. Lian Z. M. (2010). Molecular cloning, sequence analysis and expression detection of β-actin gene in the oriental armyworm, *Mythimna separata*. *Chin. Bull. Entomol.* 47 1089–1094.

[B53] Livak K. J. Schmittgen T. D. (2001). Analysis of relative gene expression data using real-time quantitative PCR and the 2(-Delta Delta C(T)) Method. *Methods* 25 402–408. 10.1006/meth.2001.1262 11846609

[B54] Lozano V. Armengaud C. Gauthier M. (2001). Memory impairment induced by cholinergic antagonists injected into the mushroom bodies of the honeybee. *J. Comp. Physiol. A* 187 249–254. 10.1007/s003590100196 11467497

[B55] Lozano V. Gauthier M. (1998). Effects of the muscarinic antagonists atropine and pirenzepine on olfactory conditioning in the honeybee. *Pharmacol. Biochem. Behav.* 59 903–907. 10.1016/s0091-3057(97)00524-8 9586847

[B56] Lü S. M. Zhao Z. Li K. Zhang Y. L. Xi G. S. (2011). Cloning and expression analysis of a muscarinic cholinergic receptor from the brain of ant, *Polyrhachis vicina*. *Arch. Insect. Biochem. Physiol.* 78 46–60. 10.1002/arch.20438 21678488

[B57] Luo J. Busillo J. M. Benovic J. L. (2008). M3 Muscarinic acetylcholine receptor-mediated signaling is regulated by distinct mechanisms. *Mol. Pharmacol.* 74 338–347. 10.1124/mol.107.044750 18388243 PMC7409535

[B58] Marchlerbauer A. Lu S. Anderson J. B. Chitsaz F. Derbyshire M. K. Deweesescott C. (2011). CDD: a Conserved Domain Database for the functional annotation of proteins. *Nucleic Acids Res.* 39:D225. 10.1093/nar/gkq1189 21109532 PMC3013737

[B59] Martin C. A. Krantz D. E. (2014). *Drosophila melanogaster* as a genetic model system to study neurotransmitter transporters. *Neurochem. Int.* 73 71–88. 10.1016/j.neuint.2014.03.015 24704795 PMC4264877

[B60] Matsuda K. Ihara M. Sattelle D. B. (2020). Neonicotinoid insecticides: molecular targets, resistance, and toxicity. *Annu. Rev. Pharmacol. Toxicol.* 60 241–255. 10.1146/annurev-pharmtox-010818-021747 31914891

[B61] Mayerhofer A. Kunz L. (2005). A non-neuronal cholinergic system of the ovarian follicle. *Ann. Anat.* 187 521–528. 10.1016/j.aanat.2005.06.005 16320831

[B62] Moro O. Lameh J. Högger P. Sadée W. (1993). Hydrophobic amino acid in the i2 loop plays a key role in receptor-G protein coupling. *J. Biol. Chem.* 268 22273–22276. 8226735

[B63] Onai T. Fitzgerald M. G. Arakawa S. Gocayne J. D. Urquhart D. A. Hall L. M. (1989). Cloning, sequence analysis and chromosome localization of a *Drosophila muscarinic* acetylcholine receptor. *FEBS Lett.* 255 219–225. 10.1016/0014-5793(89)81095-6 2507354

[B64] Reale V. Hannan F. Hall L. M. Evans P. D. (1997). Agonist-specific coupling of a cloned *Drosophila melanogaster* D1-like dopamine receptor to multiple second messenger pathways by synthetic agonists. *J. Neurosci.* 17 6545–6553. 10.1523/jneurosci.17-17-06545.1997 9254667 PMC6573129

[B65] Ren G. R. Folke J. Hauser F. Li S. Grimmelikhuijzen C. J. P. (2015). The A- and B-type muscarinic acetylcholine receptors from *Drosophila melanogaster* couple to different second messenger pathways. *Biochem. Biophys. Res. Commun.* 462 358–364. 10.1016/j.bbrc.2015.04.141 25964087

[B66] Rizzo M. J. Evans J. P. Burt M. Saunders C. J. Johnson E. C. (2018). Unexpected role of a conserved domain in the first extracellular loop in G protein coupled receptor trafficking. *Biochem. Biophys. Res. Commun.* 503 1919–1926. 10.1016/j.bbrc.2018.07.136 30064912

[B67] Robb S. Cheek T. R. Hannan F. L. Hall L. M. Midgley J. M. Evans P. D. (1994). Agonist-specific coupling of a cloned *Drosophila* octopamine/tyramine receptor to multiple second messenger systems. *EMBO J.* 13:1325. 10.1002/j.1460-2075.1994.tb06385.x 8137817 PMC394948

[B68] Ryckebusch S. Laurent G. (1993). Rhythmic patterns evoked in locust leg motor neurons by the muscarinic agonist pilocarpine. *J. Neurophysiol.* 69 1583–1595. 10.1152/jn.1993.69.5.1583 8389831

[B69] Sattelle D. B. (1980). Acetylcholine receptors of insects. *Adv. Insect Phys.* 15 215–315. 10.1016/s0065-2806(08)60142-3

[B70] Shapiro R. A. Scherer N. M. Habecker B. A. Subers E. M. Nathanson N. M. (1988). Isolation, sequence, and functional expression of the mouse M1 muscarinic acetylcholine receptor gene. *J. Biol. Chem.* 263 18397–18403. 2848036

[B71] Shapiro R. A. Wakimoto B. T. Subers E. M. Nathanson N. M. (1989). Characterization and functional expression in mammalian cells of genomic and cDNA clones encoding a *Drosophila* muscarinic acetylcholine receptor. *Proc. Natl. Acad. Sci. U.S.A.* 86 9039–9043. 10.1073/pnas.86.22.9039 2510174 PMC298428

[B72] Sidhu A. Niznik H. B. (2000). Coupling of dopamine receptor subtypes to multiple and diverse G proteins. *Int. J. Dev. Neurosci.* 18 669–677. 10.1016/s0736-5748(00)00033-2 10978845

[B73] Silva B. Molina-Fernández C. Ugalde M. B. Tognarelli E. I. Angel C. Campusano J. M. (2015). Muscarinic ACh receptors contribute to aversive olfactory learning in *Drosophila*. *Neural Plast.* 2015:658918. 10.1155/2015/658918 26380118 PMC4562076

[B74] Smith R. D. Grzelak M. E. Coffin V. L. (1994). Methylatropine blocks the central effects of cholinergic antagonists. *Behav. Pharmacol.* 5 167–175. 10.1097/00008877-199404000-00008 11224265

[B75] Thomas P. Smart T. G. (2005). HEK293 cell line: a vehicle for the expression of recombinant proteins. *J. Pharmacol. Toxicol. Methods* 51 187–200. 10.1016/j.vascn.2004.08.014 15862464

[B76] Torkkeli P. H. Widmer A. Meisner S. J. (2005). Expression of muscarinic acetylcholine receptors and choline acetyltransferase enzyme in cultured antennal sensory neurons and non-neural cells of the developing moth *Manduca sexta*. *Neurobiology* 62 316–329. 10.1002/neu.20097 15514997

[B77] Trimmer B. A. (1995). Current excitement from insect muscarinic receptors. *Trends Neurosci.* 18 104–111. 10.1016/0166-2236(95)80032-w 7537401

[B78] von der Kammer H. Mayhaus M. Albrecht C. Enderich J. Wegner M. Nitsch R. M. (1998). Muscarinic acetylcholine receptors activate expression of the EGR gene family of transcription factors. *J. Biol. Chem.* 273 14538–14544. 10.1074/jbc.273.23.14538 9603968

[B79] Wessler I. Gärtner H. A. Michel-Schmidt R. Brochhausen C. Schmitz L. Anspach L. (2016). Honeybees produce millimolar concentrations of non-neuronal acetylcholine for breeding: possible adverse effects of neonicotinoids. *PLoS One* 11:e0156886. 10.1371/journal.pone.0156886 27285384 PMC4902251

[B80] Wessler I. Kirkpatrick C. J. (2008). Acetylcholine beyond neurons: the non-neuronal cholinergic system in humans. *Br. J. Pharmacol.* 154 1558–1571. 10.1038/bjp.2008.185 18500366 PMC2518461

[B81] Wessler I. Kirkpatrick C. J. (2017). Non-neuronal acetylcholine involved in reproduction in mammals and honeybees. *J. Neurochem.* 142 144–150. 10.1111/jnc.13953 28072454

[B82] Wessler I. Kirkpatrick C. J. Racké K. (1998). Non-neuronal acetylcholine, a locally acting molecule, widely distributed in biological systems: expression and function in humans. *Pharmacol. Ther.* 77 59–79. 10.1016/s0163-7258(97)00085-5 9500159

[B83] Wessler I. Kirkpatrick C. J. Racké K. (1999). The cholinergic ‘pitfall’: acetylcholine, a universal cell molecule in biological systems, including humans. *Clin. Exp. Pharmacol. Physiol.* 26 198–205. 10.1046/j.1440-1681.1999.03016.x 10081614

[B84] Xia R. Y. Li M. Q. Wu Y. S. Qi Y. X. Ye G. Y. Huang J. (2016). A new family of insect muscarinic acetylcholine receptors. *Insect. Mol. Biol.* 25 362–369. 10.1111/imb.12229 27003873

[B85] Yoshihara M. Ensminger A. W. Littleton J. T. (2001). Neurobiology and the *Drosophila* genome. *Funct. Integr. Genomics* 1 235–240. 11793242 10.1007/s101420000029

